# Bcl-2 family inhibition sensitizes human prostate cancer cells to docetaxel and promotes unexpected apoptosis under caspase-9 inhibition

**DOI:** 10.18632/oncotarget.2550

**Published:** 2014-10-15

**Authors:** Hiroki Tamaki, Nanae Harashima, Miho Hiraki, Naoko Arichi, Nobuhiro Nishimura, Hiroaki Shiina, Kohji Naora, Mamoru Harada

**Affiliations:** ^1^ Department of Immunology, Shimane University Faculty of Medicine, Shimane, Japan; ^2^ Department of Pharmacy, Shimane University Hospital, Shimane, Japan; ^3^ Department of Urology, Shimane University Faculty of Medicine, Shimane, Japan

**Keywords:** prostate cancer, docetaxel, apoptosis, Bcl-2, Bcl-xL

## Abstract

Docetaxel (DTX) is a useful chemotherapeutic drug for the treatment of hormone-refractory prostate cancer. However, emergence of DTX resistance has been a therapeutic hurdle. In this study, we investigated the effect of combining DTX with Bcl-2 family inhibitors using human prostate cancer cell lines (PC3, LNCaP, and DU145 cells). PC3 cells were less sensitive to DTX than were the other two cell lines. In contrast to ABT-199, which inhibits Bcl-2 and Bcl-w, both ABT-263 and ABT-737, which inhibit Bcl-2, Bcl-xL, and Bcl-w, significantly augmented the antitumor effect of DTX on PC3 cells. ABT-263 also enhanced the antitumor effect of DTX on a DTX-resistant PC3 variant cell line. The antitumor effect of ABT-263 was due mainly to its inhibitory effect on Bcl-xL. In a xenograft mouse model, DTX and ABT-737 combination therapy significantly inhibited PC3 tumor growth. Interestingly, although ABT-263 activated caspase-9 in PC3 cells, inhibition of caspase-9 unexpectedly promoted ABT-263-induced apoptosis in a caspase- 8-dependent manner. This augmented apoptosis was also observed in LNCaP cells. These findings indicate that Bcl-xL inhibition can sensitize DTX-resistant prostate cancer cells to DTX, and they reveal a unique apoptotic pathway in which antagonism of Bcl-2 family members in caspase-9-inhibited prostate cancer cells triggers caspase-8-dependent apoptosis.

## INTRODUCTION

Prostate cancer is one of the most common malignant disorders found in males worldwide. Although early-stage prostate cancer can be well-controlled by surgery or radiotherapy, patients with advanced prostate cancer are treated with hormone therapy [1], and after a short-term remission, surviving cancer cells often return with increased malignancy [2]. Docetaxel (DTX) has been used as a chemotherapeutic drug to combat recurrent prostate cancer [3–5]; however, malignant cells frequently acquire DTX resistance, and efficient treatment modalities to overcome this resistance are required.

Apoptosis is primarily induced in cancer cells through two major pathways: extrinsic and intrinsic pathways [6, 7]. Fas ligand (FasL) and tumor necrosis factor (TNF)-related apoptosis-inducing ligand (TRAIL) can provide a death signal via the ‘extrinsic’ apoptotic pathway, activating caspase-8 in cancer cells. In contrast, cytotoxic drugs and high-dose radiation damage DNA and mitochondria, resulting in activation of the ‘intrinsic’ caspase-9-mediated apoptotic pathway. Although several molecules participate in mitochondria-mediated apoptosis [8–10], Bcl-2 family molecules play a crucial role in this type of apoptosis [11, 12]. The family of Bcl-2-related anti-apoptotic proteins includes Bcl-2, Bcl-xL, Bcl-w, and Mcl-1. These proteins inhibit cell death by sequestering the pro-apoptotic proteins Bax and Bak and by preventing their oligomerization [13–16]. Elevation of Bcl-2 expression protects cancer cells from apoptosis [17, 18], and the elevated expression of Bcl-2 and Bcl-xL has been frequently observed in a variety of cancers [12]. Additionally, a survey of gene expression and response to chemotherapy agents in the NCI-60 panel identified Bcl-xL as a major cause of chemoresistance in epithelial cancer cells [19]. Thus, inhibition of Bcl-2 and/or Bcl-xL is hypothesized to potentiate the effect of chemotherapy, and consequently several Bcl-2 family inhibitors/antagonists have been developed. ABT-737 is a small molecule inhibitor of Bcl-2, Bcl-xL, and Bcl-w [20]. ABT-263 (Navitoclax) is a clinically available and orally bioavailable inhibitor with the same specificity as ABT-737 [21, 22]. In addition, ABT-199 is a new, orally bioavailable inhibitor that inhibits Bcl-2 and Bcl-w, but not Bcl-xL [23]. Several reports have shown efficacy of these inhibitors against both hematological malignancies as well as several types of solid tumors [24–30].

In this study, we investigated the effect of combining DTX with Bcl-2 family inhibitors in three human prostate cancer cell lines: PC3, LNCaP, and DU145 cells. Among them, PC3 cells were less sensitive to DTX than were the other two lines, but ABT-263 and ABT-737 significantly augmented the sensitivity of these cells to DTX. RNA interference experiments showed that ABT-263 augmented the antitumor effect of DTX on PC3 cells via Bcl-xL inhibition. In a xenograft mouse model, DTX and ABT-737 combination therapy significantly inhibited the growth of PC3 cells compared with either therapy alone. Additionally, despite the fact that ABT-263 activated caspase-9 in PC3 cells, inhibition of caspase-9 unexpectedly promoted ABT-263-induced apoptosis in a caspase-8-dependent manner. These findings indicate that Bcl-xL inhibition by ABT-263 or ABT-737 can sensitize DTX-resistant prostate cancer cells to DTX, and they reveal a unique apoptotic pathway in which antagonism of Bcl-2 family members in caspase-9-inhibited prostate cancer cells triggers caspase-8-dependent apoptosis.

## RESULTS

### The therapeutic effect of combining DTX with Bcl-2 family inhibitors in human prostate cancer cells

**Figure 1:**
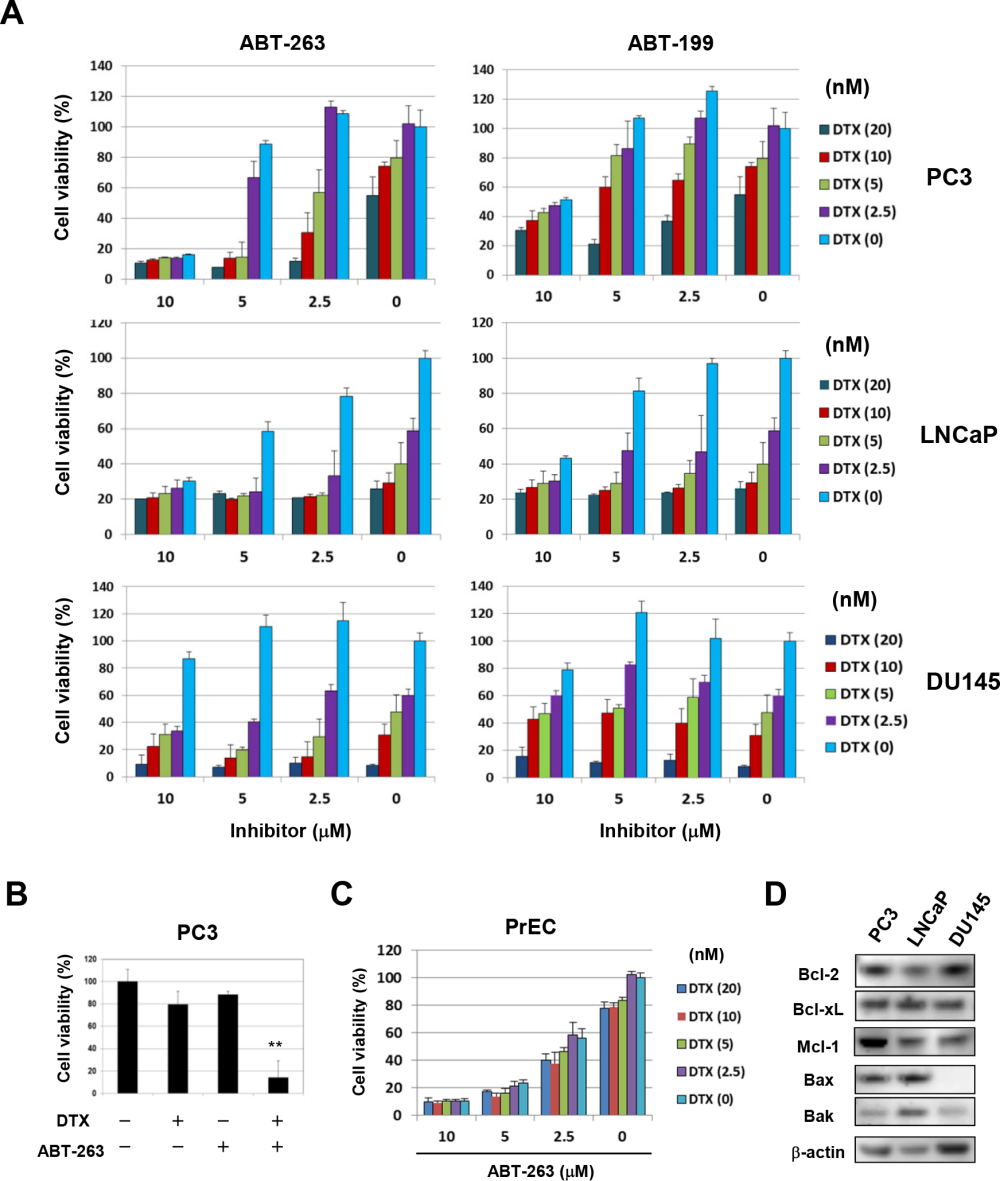
ABT-263 sensitizes PC3 cells to DTX **(A)** Three prostate cancer cell lines were cultured with the indicated concentrations of DTX (nM) and either ABT-263 or ABT-199 (μM). After 48 h, cell viability (%) was determined using the WST-8 assay. The results are shown as the means + SD of three wells. Similar results were obtained in three independent experiments. **(B)** Selected results for DTX (5 nM) and ABT-263 (5 μM) treatments are shown. **P<0.01 compared with the other three groups (Student's *t*-test). **(C)** Normal prostate epithelial PrEC cells were cultured with the indicated concentrations of DTX and ABT-263, and cell viability (%) was determined as described above. **(D)** The three prostate cancer cell lines were examined for their expression of Bcl-2 family molecules by immunoblot. β-actin was used as the control. DTX, docetaxel.

Initially, the cytotoxic effect of combining DTX with either of two Bcl-2 family inhibitors, ABT-263 and ABT-199, was assessed using three prostate cancer cell lines (Fig. 1A). Among the three cell lines, PC3 cells were relatively resistant to DTX and DU145 cells were less sensitive to both inhibitors compared with the other two cell lines. Of note, ABT-263 decreased the viability of PC3 cells more drastically than did ABT-199 with suboptimal doses of DTX. Selected data are shown in Fig. 1B. Such a synergistic effect was not observed in LNCaP or DU145 cells. The effect of these drug combinations on a normal prostate epithelial cell line, PrEC, was also assessed (Fig. 1C). PrEC cells were less sensitive to DTX but more sensitive to ABT-263, compared with prostate cancer cell lines. No synergistic effect of DTX combined with ABT-263 was observed in PrEC cells. The expression of Bcl-2 family proteins (anti-apoptotic proteins including Bcl-2, Bcl-xL, and Mcl-1 and pro-apoptotic proteins including Bax and Bak) was then examined in the three prostate cancer cell lines (Fig. 1D). All three cell lines showed expression of each molecule evaluated at the protein level, except for a lack of detectable expression of Bax in DU145 cells.

### Bcl-xL plays a major role in protection of PC3 cells against DTX cytotoxicity

**Figure 2:**
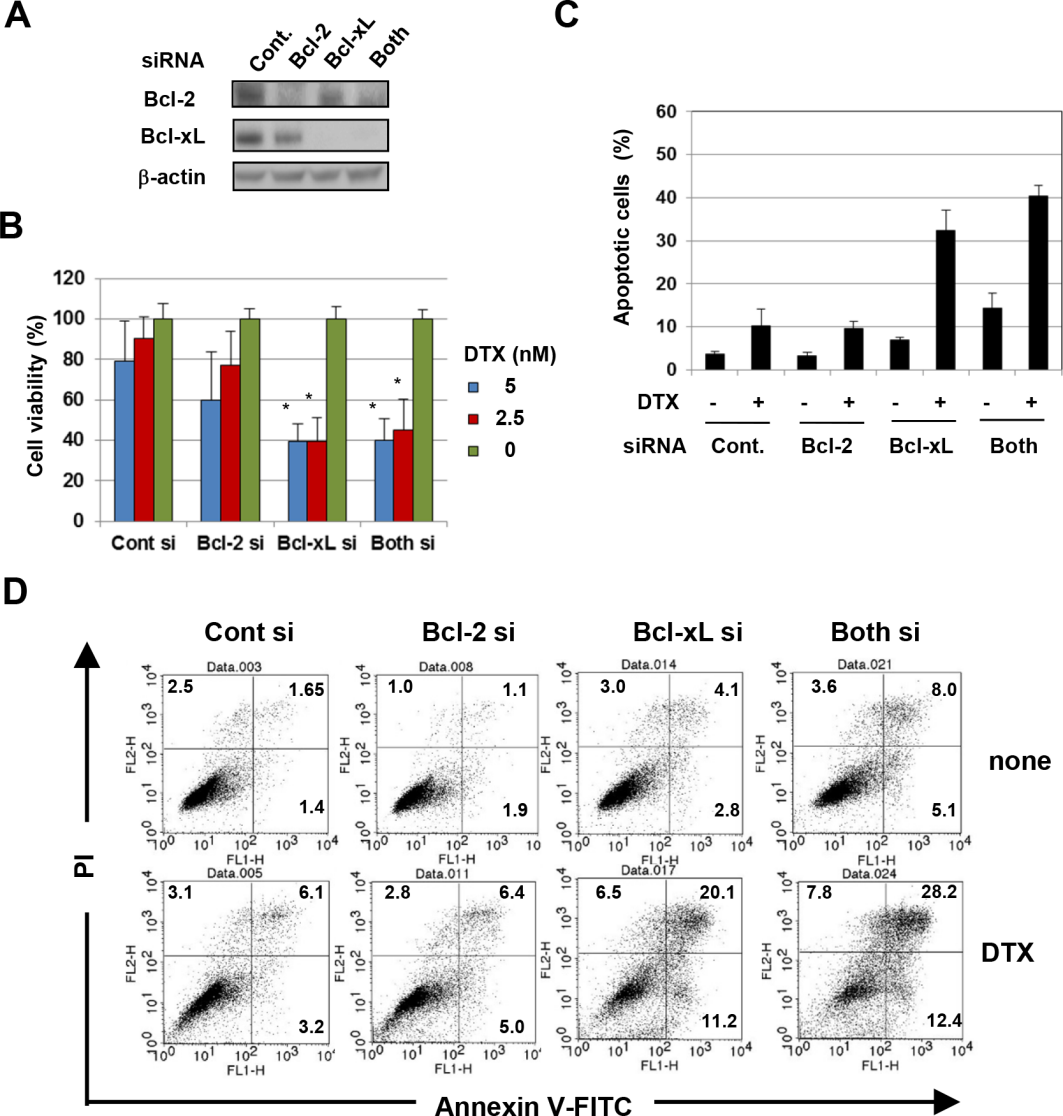
The antitumor effect of ABT-263 on PC3 cells is mainly due to inhibition of Bcl-xL **(A)** PC3 cells transfected with the indicated siRNA were analyzed for their expression of Bcl-2 and Bcl-xL by immunoblot. β-actin was used as the control. **(B)** siRNA-transfected PC3 cells were cultured with the indicated concentrations of DTX (nM). After 48 h, cell viability (%) was determined using the WST-8 assay. The results are shown as the means + SD of three wells. **(C)** siRNA-transfected PC3 cells were cultured with DTX (5 nM). After 24 h, cells were stained with FITC-conjugated Annexin V/PI, and flow cytometric analysis was performed. The results are shown as the means + SD of three wells. **P<0.01 (Student's *t*-test) **(D)** Representative results are shown. The numbers represent the percentages of each subset. DTX, docetaxel.

Given the difference in specificity of inhibition between ABT-263 and ABT-199, we examined whether the augmenting effect of ABT-263 was due to its inhibition of Bcl-xL alone versus the inhibition of both Bcl-xL and Bcl-2. When transfected into PC3 cells, small interfering RNA (siRNA) specific to either Bcl-2 or Bcl-xL decreased the expression of the respective proteins (Fig. 2A). Additionally, although selective knockdown of Bcl-2 showed a tendency to decrease cell viability, Bcl-xL knockdown significantly decreased the viability of PC3 cells in the presence of low-dose DTX, to the same level as that caused by double knockdown of Bcl-2 and Bcl-xL (Fig. 2B). Relatedly, knockdown of Bcl-xL increased the percentage of apoptotic PC3 cells in the presence of low-dose DTX to the same level as that caused by double knockdown of Bcl-2 and Bcl-xL (Fig. 2C and D). These results indicate that the augmented antitumor effect induced by ABT-263 in PC3 cells treated with low-dose DTX is primarily due to inhibition of Bcl-xL.

### Effect of combining DTX with ABT-263 in a DTX-resistant PC3 variant cell line

**Figure 3:**
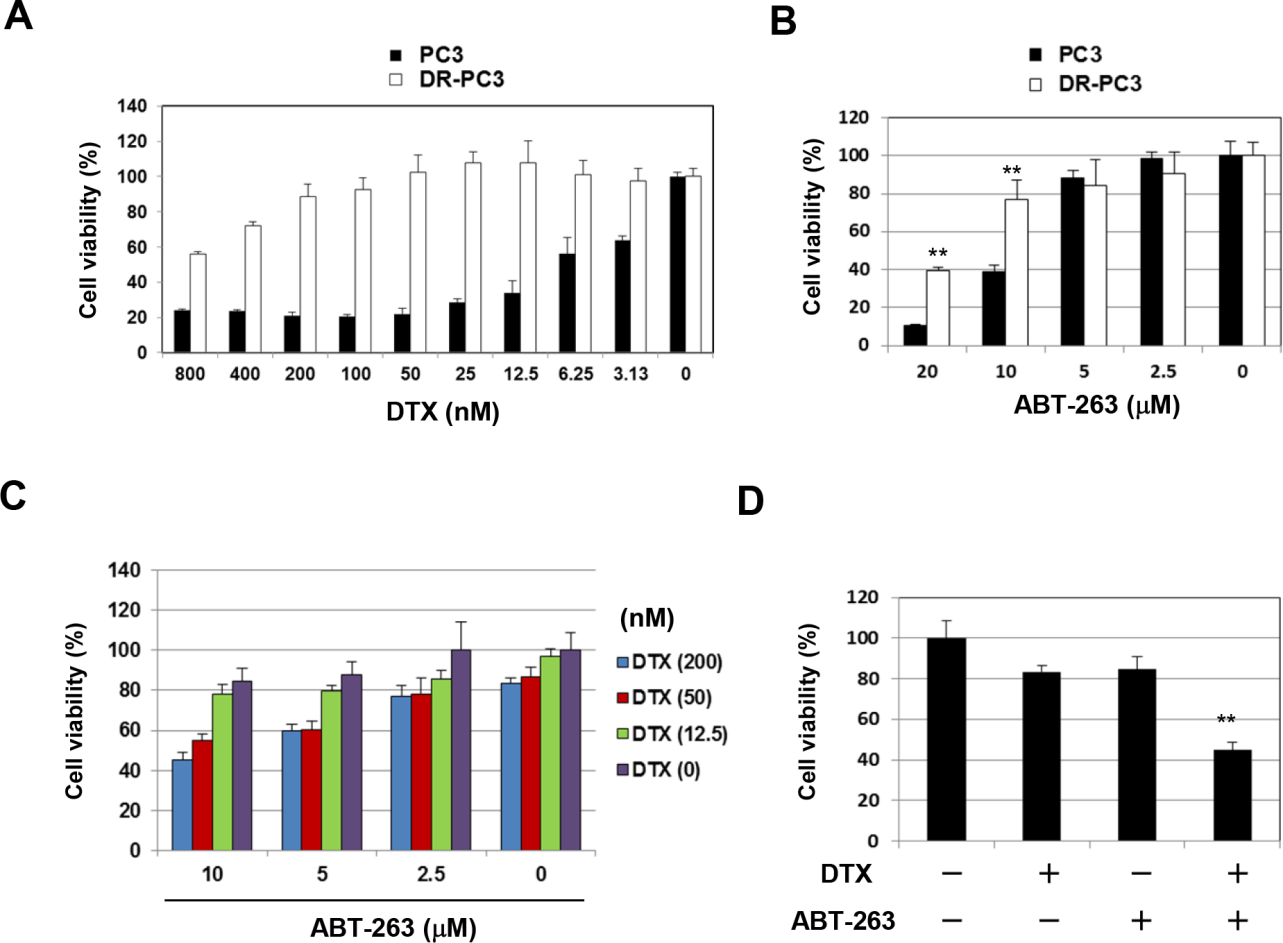
ABT-263 sensitizes a DTX-resistant PC3 variant cell line to DTX **(A)** Parental PC3 and DR-PC3 cells were cultured with the indicated concentrations of DTX (nM). After 48 h, cell viability (%) was assessed using the WST-8 assay. The results are shown as the means + SD of three wells. **(B)** Similarly, PC3 and DR-PC3 cells were examined for their sensitivity to ABT-263 (μM). **P<0.01 (Student's *t*-test). **(C)** DR-PC3 cells were cultured with the indicated concentrations of DTX (nM) and/or ABT-263 (μM). After 48 h, cell viability (%) was determined as described above. **(D)** Selected results are shown, as means + SD of three wells. **P<0.01 compared with the other three groups (Student's *t*-test). DTX, docetaxel.

As described above, the emergence of DTX resistance in prostate cancer cells has become an important therapeutic challenge. Accordingly, we established a DTX-resistant PC3 variant cell line, designated DR-PC3, and examined the effect of DTX and ABT-263 combination treatment in this cell line. DR-PC3 cells grew more slowly than did parental PC3 cells and showed a thinner appearance than the parental PC3 cells (data not shown). The DR-PC3 cells showed significant resistance to DTX compared with parental PC3 cells (Fig. 3A). DR-PC3 cells also exhibited higher resistance to ABT-263 at a dose of 10 or 20 μM, compared with parental PC3 cells (Fig. 3B). When treated with both DTX and ABT-263, although the sensitizing effect was not as dramatic as seen in parental PC3 cells, ABT-263 (5 or 10 μM) augmented the antitumor effect of 50 and 200 nM DTX (Fig. 3C). Selected data are shown in Fig. 3D. These results suggest that ABT-263 has the potential to sensitize DTX-resistant prostate cancer cells to DTX-induced cytotoxicity.

### The effect of DTX and ABT-737 co-treatment on PC3 cells in a xenograft mouse model

**Figure 4:**
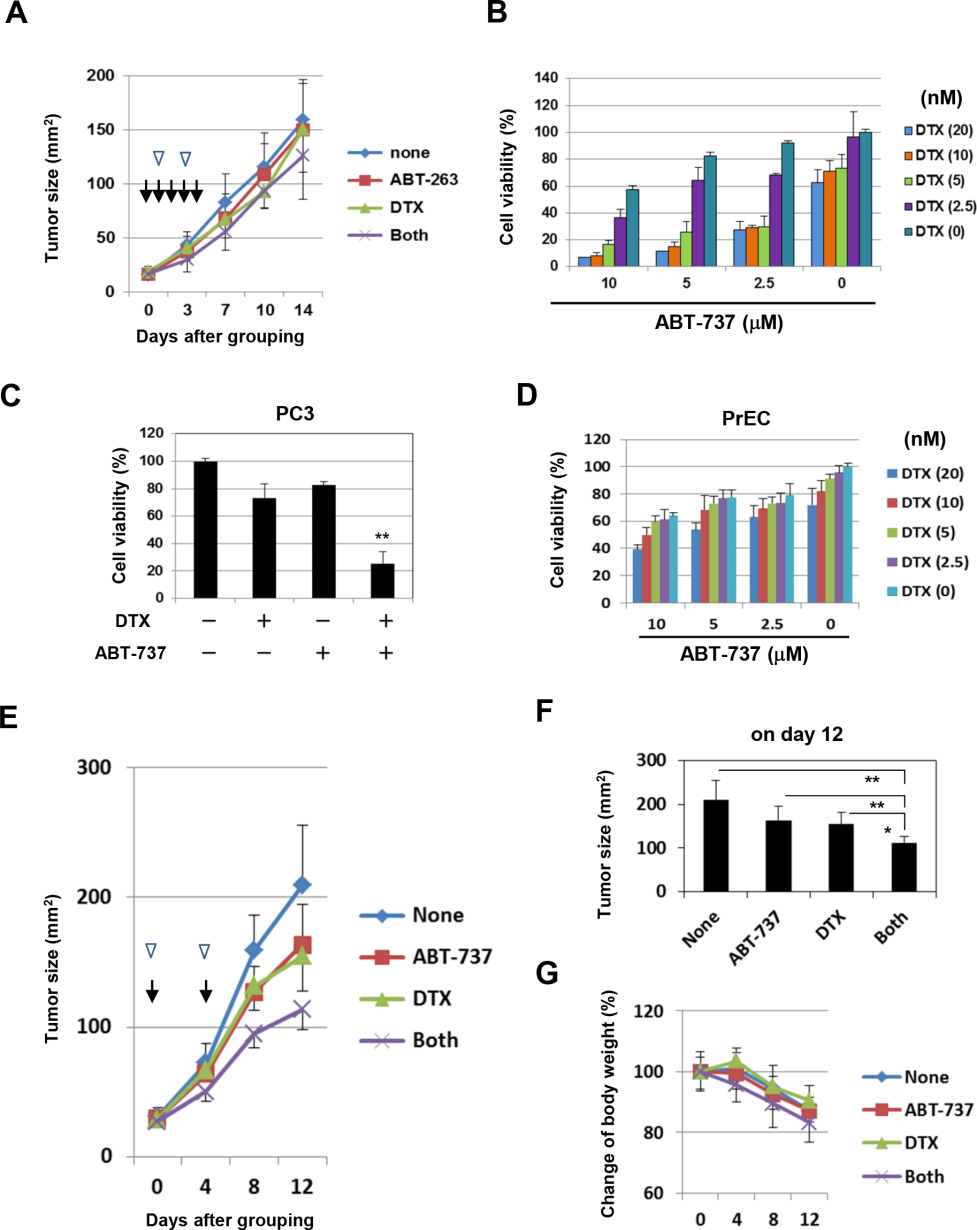
*In vivo* antitumor effect of DTX and ABT-737 on the growth of PC3 cells **(A)** BALB *nu/nu* male mice (n = 6) were inoculated in the right flank with 3 × 10^6^ PC-3 cells in Matrigel. On day 7, the mice were pooled and divided into four groups. The PC3-bearing mice were administered with either or both DTX (10 mg/kg) intraperitoneally on days 1 and 3 (arrow heads) and ABT-263 (20 mg/kg) orally on days 0, 1, 2, 3, and 4 (arrows) after grouping. Thereafter, the tumor size, product of two perpendicular diameters, was measured every 3 or 4 days. The results are shown as the means + SD of six mice. **(B)** PC3 cells were cultured with the indicated concentrations of DTX (nM) and ABT-737 (μM). After 48 h, cell viability (%) was assessed using the WST-8 assay. The results are shown as the means + SD of three wells. **(C)** Selected results are shown, as the means + SD of three wells. **P<0.01 compared with the other three groups (Student's *t*-test) **(D)** PrEC cells were cultured with the indicated concentrations of DTX and ABT-263, and cell viability (%) was determined as described above. **(E, F)** BALB *nu/nu* male mice (n = 6) were inoculated in the right flank with 3 × 10^6^ PC-3 cells in Matrigel. On day 7, the mice were pooled and divided into four groups. On days 0 and 4 after grouping, the PC3-bearing mice were injected intraperitoneally with either or both DTX (10 mg/kg) (arrow heads) and ABT-737 (50 mg/kg) (arrows). Thereafter, the tumor size, product of two perpendicular diameters, and body weight **(G)** were measured every 4 days. The results are shown as the means + SD of six mice. *P<0.05, **P<0.01 (ANOVA with Bartlett's test). DTX, docetaxel.

We next examined the antitumor effect induced by the combination of DTX and Bcl-2 family inhibitors *in vivo*. Initially, ABT-263 (20 mg/kg) was administered orally on days 0, 1, 2, 3, and 4 after grouping in combination with DTX (10 mg/kg) injected intraperitoneally (i.p.) on days 1 and 3 after grouping; no significant synergistic effect on the growth of PC3 was observed (Fig. 4A). Subsequently, we used ABT-737, which has the same specificity as ABT-263 yet can be administered systemically. In *in vitro* studies, the combination of ABT-737 and DTX synergistically decreased the viability of PC3 cells to a similar degree as seen with ABT-263 (Fig. 4B and C). ABT-737 showed a similar effect on the normal prostate epithelial cell line PrEC, but to a lesser degree than that of ABT-263 (Fig. 4D). To determine the doses of DTX and ABT-737 used for *in vivo* study, we performed preliminary experiments. In the first, all PC3-bearing mice died following i.p. administration of DTX (30 mg/kg) on days 0, 2, and 4 after grouping, suggesting that DTX (30 mg/kg) administration three times at 2-day intervals was too much. In the second experiment, although i.p. administration of DTX (10 mg/kg) or ABT-737 (100 mg/kg) alone on days 0, 3, and 6 after grouping showed no effect on mortality, the combination of both resulted in the deaths of all of the mice. Based on these results, we performed experiments in which PC3-bearing mice were injected i.p. with DTX (10 mg/kg) and/or ABT-737 (50 mg/kg) on days 0 and 4 after grouping (Fig. 4E). In PC3-grafted nude mice, DTX and ABT-737 combination treatment significantly suppressed tumor growth compared with the groups treated with either drug alone (Fig. 4E and F). Body weight was also measured, as an indicator of general health, and was found to decrease in all groups, in accompaniment with tumor growth and probably due to cachexia. Body weight loss was most apparent in the mice treated with the combination therapy, but the difference was not significant, and no mortality was observed (Fig. 4G). These results indicate that Bcl-2 family inhibitors such as ABT-737 can sensitize the partially DTX-resistant human prostate cancer cells to DTX *in vivo*.

### Induction of caspase-dependent apoptosis in PC3 cells by co-treatment with DTX and ABT-263

**Figure 5:**
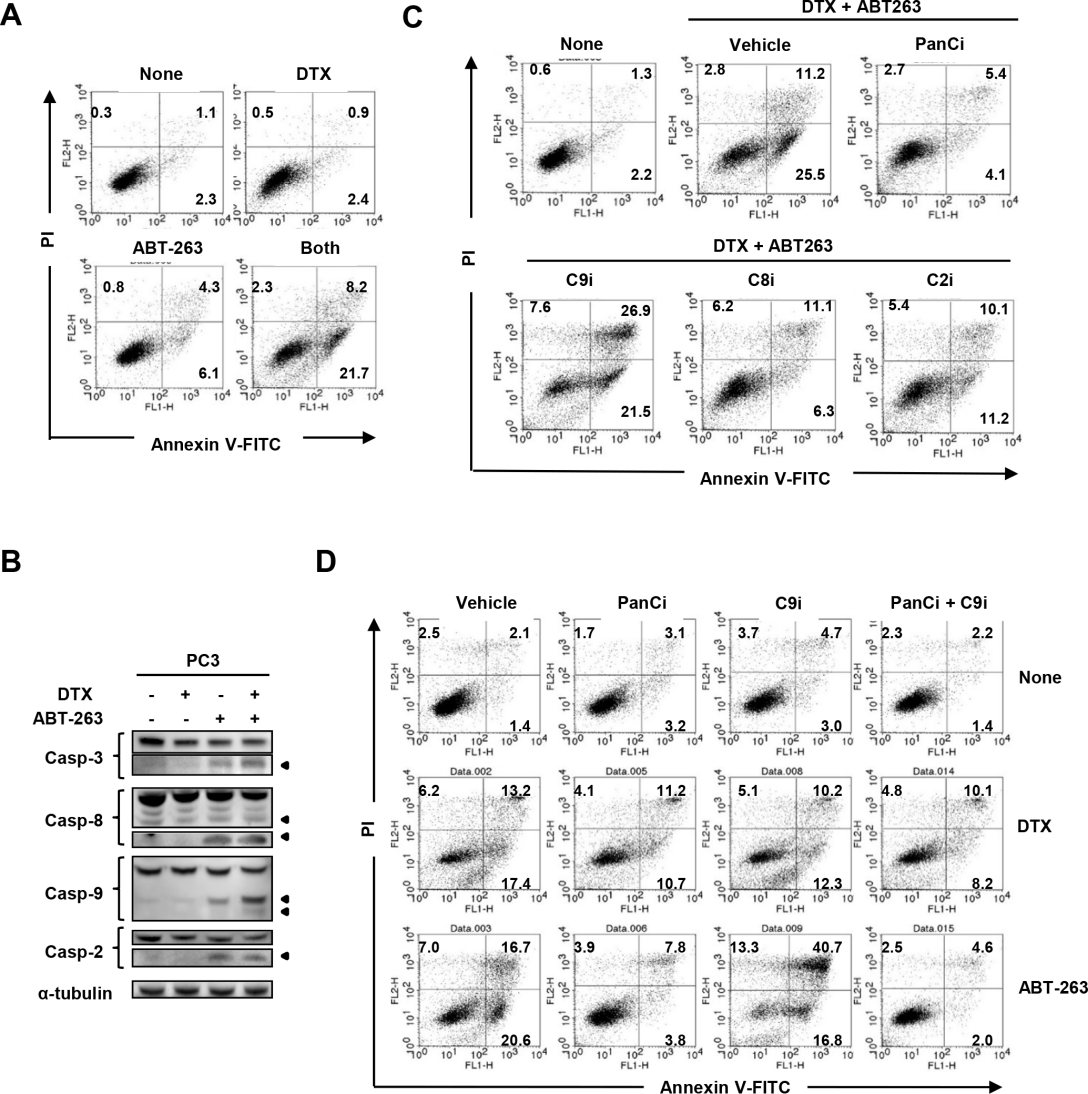
Inhibition of caspase-9 promotes apoptosis in ABT-263-treated PC3 cells **(A)** PC3 cells were treated with both DTX (2.5 nM) and ABT-263 (4 μM). After 24 h, cells were stained with FITC-conjugated Annexin V and PI, and flow cytometry was performed. The numbers represent the percentages of each subset. **(B)** PC3 cells were treated with DTX (2.5 nM) and/or ABT-263 (4 μM). After 24 h, cells were harvested and cell lysates assayed for their expression of caspase-3, -8, -9, and -2 by immunoblot. β-actin was used as a loading control. **(C)** PC3 cells were treated with both DTX (2.5 nM) and ABT-263 (4 μM) in the presence of the indicated caspase inhibitors. After 24 h, flow cytometry was performed as described previously. The numbers represent the percentages of each subset. **(D)** PC3 cells were treated with either DTX (2.5 nM) or ABT-263 (4 μM) in the presence of the indicated caspase inhibitors. DTX, docetaxel; panCi, pan-caspase inhibitor; C9i, caspase-9 inhibitor; C8i, caspase-8 inhibitor; C2i, caspase-2 inhibitor. As the vehicle control, the same volume of DMSO was added.

To examine the mechanism underlying the synergistic antitumor effect of DTX and ABT-263, flow cytometric analysis of Annexin V/propidium iodide (PI) was performed. As shown in Fig. 5A, treatment of PC3 cells with the combination therapy increased the proportion of Annexin V^+^ apoptotic cells significantly, as compared with either therapy alone. Immunoblot analysis revealed that treatment of PC3 cells with ABT-263 alone activated caspase-3, -8, -9, and -2, and that co-treatment with DTX further increased the activation levels of caspase-3 and -9 (Fig. 5B), implying that combination therapy enhanced apoptosis in a caspase-9-dependent manner. This phenomenon was further confirmed using a panel of caspase inhibitors. In PC3 cells co-treated with DTX and ABT-263, the percentage of Annexin V^+^ apoptotic cells was decreased by the addition of inhibitors against pan-caspase, caspase-8, or caspase-2 (Fig. 5C). Unexpectedly, incubation with a caspase-9 inhibitor increased the percentage of Annexin V^+^ apoptotic PC3 cells. The degree of apoptosis was further enhanced when caspase-9 inhibitor-treated PC3 cells were co-treated with ABT-263, but not with DTX (Fig. 5D).

### Caspase-8-dependent apoptosis in ABT-263-treated PC3 and LNCaP cells is augmented by caspase-9 inhibition

**Figure 6:**
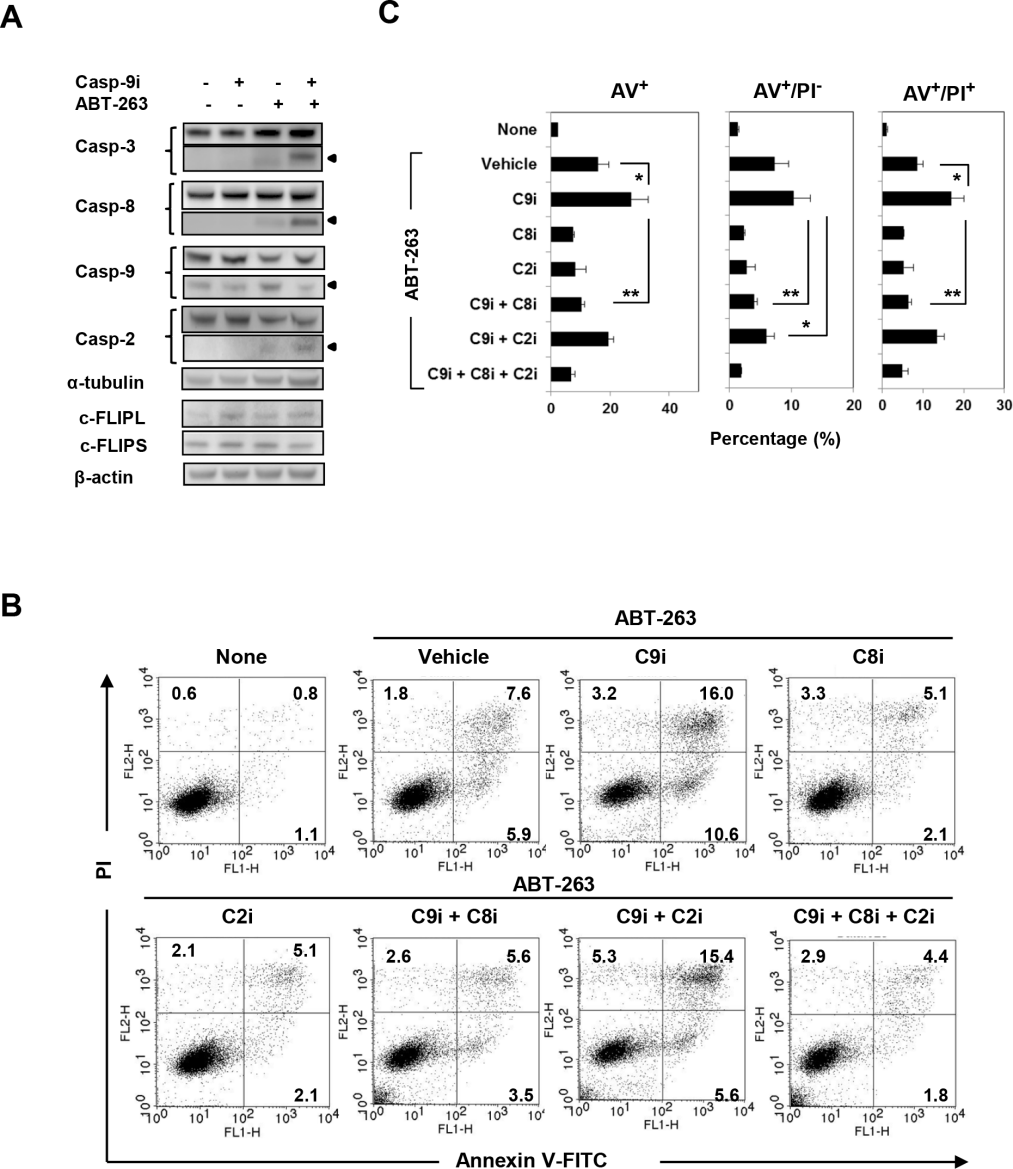
Analysis of caspase-8-dependent ABT-263-induced apoptosis of PC3 cells under caspase-9 inhibition **(A)** PC3 cells were treated with a caspase-9 inhibitor (20 μM) and/or ABT-263 (4 μM). After 24 h, cells were harvested and cell lysates assayed for their expression of caspase-3, -8, -9, -2, and c-FLIP by immunoblot. α-tubulin and β-actin were used as loading controls. **(B)** PC3 cells were treated with both DTX (2.5 nM) and ABT-263 (4 μM) in the presence of the indicated caspase inhibitors. After 24 h, cells were stained with FITC-conjugated Annexin V and PI, and flow cytometry was performed. The numbers represent the percentages of each subset. **(C)** The results are shown as the means + SD of three samples. *P<0.05, **P<0.01 (Student's *t*-test); panCi, pan-caspase inhibitor; C9i, caspase-9 inhibitor; C8i, caspase-8 inhibitor; C2i, caspase-2 inhibitor. As the vehicle control, the same volume of DMSO was added.

We next explored the mechanism by which ABT-263-induced apoptosis was augmented by incubation with a caspase-9 inhibitor. As shown in Fig. 6A, treatment with ABT-263 alone activated mainly caspase-9 in PC3 cells, whereas co-treatment with the caspase-9 inhibitor clearly enhanced activation of caspase-3 and caspase-8. Caspase-2 activation was also enhanced slightly. Since cellular FLICE-like inhibitory proteins (c-FLIPs) are known to inhibit caspase-8 activation [6, 31], we examined the expression of c-FLIP_L_ and c-FLIP_S_ in treated PC3 cells. However, no change in cFLIP expression was observed in cells treated with both the caspase-9 inhibitor and ABT-263 (Fig. 6A). We confirmed this phenomenon by flow cytometry. Augmentation of Annexin V^+^/PI^−^ (early) and Annexin V^+^/PI^+^ (late) apoptosis of ABT-263-treated PC3 cells induced by the caspase-9 inhibitor was clearly blocked by caspase-8 inhibition (Fig. 6B and C). Caspase-2 inhibition also blocked the enhanced apoptosis (Annexin V^+^/PI^−^) observed in ABT-263/caspase-9 inhibitor co-treated PC3 cells, but the restoration effect was small.

Given the existence of the caspase-9-independent, Smac-mediated mitochondrial apoptotic pathway [32, 33], the involvement of Smac in the enhanced apoptosis observed was examined. However, knockdown of Smac by siRNA failed to affect the increase in apoptosis seen in the presence of both ABT-263 and the caspase-9 inhibitor (Suppl. Fig. 1). Given that apoptosis can also be induced in human prostate cancer cells via the interaction of Fas and FasL [34, 35], we examined the involvement of this pathway in the augmented, caspase-8-dependent apoptosis of PC3 cells co-treated with ABT-263 and the caspase-9 inhibitor. However, PC3 cells showed no Fas expression and only a portion of the cells expressed FasL; furthermore, incubation with an anti-FasL blocking antibody had no effect on apoptosis of PC3 cells treated with both ABT-263 and the caspase-9 inhibitor (Suppl. Fig. 2).

**Figure 7:**
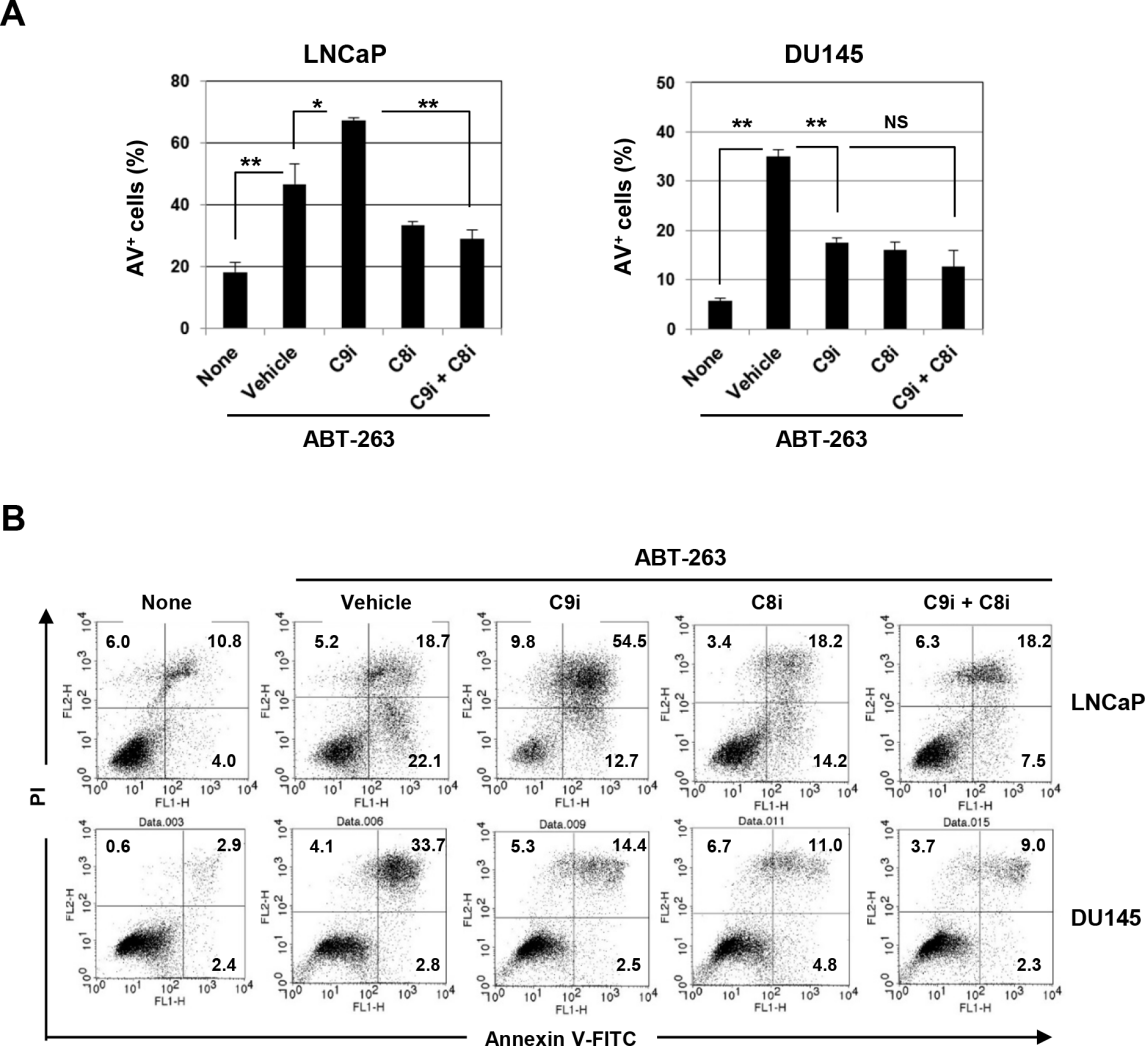
Induction of caspase-8-dependent ABT-263-induced apoptosis of LNCaP cells by inhibition of caspase-9 **(A)** LNCaP and DU145 cells were treated with ABT-263 (2.5 μM for LNCaP and 10 μM for DU145) in the presence of either or both the caspase-9 and caspase-8 inhibitors (20 μM). After 24 h, cells were stained with FITC-conjugated Annexin V and PI, and flow cytometry was performed. The results are shown as the means + SD of three samples. *P<0.05, **P<0.01 (Student's *t*-test) NS, not significant. **(B)** Representative results are shown. The numbers represent the percentages of each subset. C9i, caspase-9 inhibitor; C8i, caspase-8 inhibitor. As the vehicle control, the same volume of DMSO was added.

The effect of ABT-263/caspase-9 inhibitor co-treatment on apoptosis was also examined in several other prostate cancer cell lines. Caspase-9 inhibition was found to significantly increase apoptosis in ABT-263-treated LNCaP cells, and subsequent inhibition of caspase-8 blocked this augmentation (Fig. 7A). In contrast, the addition of the caspase-9 inhibitor significantly inhibited apoptosis of ABT-263-treated DU145 cells. Representative data are shown in Fig. 7B.

## DISCUSSION

Prostate cancer has become an increasingly prevalent malignancy in males worldwide. Although several treatment options exist for patients with prostate cancer, DTX resistance is an urgent issue to be overcome as quickly as possible. In this study, we investigated the therapeutic effect of combining DTX and Bcl-2 family inhibitors in human prostate cancer cells. Our findings revealed that ABT-263 and ABT-737 have the potential to enhance the sensitivity of the partially DTX-insensitive PC3 prostate cancer cell line, and that this effect is largely due to Bcl-xL inhibition. Additionally, we uncovered a unique apoptotic pathway in which ABT-263 and caspase-9 inhibition paradoxically promote apoptosis in human prostate cancer cells, despite the fact that ABT-263 activates caspase-9.

Of the three human prostate cancer cell lines examined, PC3 cells were less sensitive to DTX, compared with LNCaP and DU145 cells, and exhibited enhanced sensitivity to DTX when treated with ABT-263, but not with ABT-199 (Fig. 1). Given the difference in specificity between ABT-263 and ABT-199, we examined whether the augmenting effect of ABT-263 was dependent on inhibition of Bcl-xL alone versus both Bcl-xL and Bcl-2. RNA interference experiments revealed that Bcl-xL inhibition by ABT-263 was mainly responsible for the augmented cytotoxicity in PC3 cells (Fig. 2). This result was plausible because cytotoxic anti-cancer drugs induce mitochondria-mediated and caspase-9-dependent apoptosis and because Bcl-xL over-expression is a major cause of chemoresistance in many types of epithelial cancer cells [19].

Apoptosis is triggered primarily through the ‘extrinsic’ or ‘intrinsic’ caspase-dependent cell death pathways, and caspase-8 and -9, respectively, play central roles in these pathways [6, 7]. Additionally, activation of caspase-8 transforms Bid to tBid, thereby promoting mitochondrial-mediated caspase-9-dependent apoptosis. When cancer cells are exposed to anti-cancer drugs, changes in pro-apoptotic or anti-apoptotic molecules occur at the mitochondrial membrane, leading to activation of caspase-9 [6]. In the current study, although sub-optimal doses of DTX failed to activate any caspases, ABT-263 alone activated caspase-3, -8, -9, and -2 in PC3 cells (Fig. 5B). The combination of DTX and ABT-263 further increased the activation of caspase-3 and -9, suggesting that the enhanced apoptotic effect achieved by co-treatment with DTX and ABT-263 was due mainly to caspase-9 activation.

Unexpectedly, although ABT-263 induced caspase-9 activation, apoptosis of ABT-263-treated PC3 cells was paradoxically increased by the addition of a caspase-9 inhibitor. Of note, this augmented apoptosis was inhibited by the pan-caspase inhibitor z-VAD (Fig. 5D), suggesting that the enhanced apoptosis was definitely caspase-dependent. Therefore, we attempted to elucidate the underlying mechanism. In addition to the caspase-9-mediated pathway, the release of Smac/DIABLO from the mitochondria promotes caspase-dependent apoptosis by inhibiting apoptotic inhibitors [32, 33]. Therefore, this possibility was examined; however, knockdown of Smac had no effect on the induction of apoptosis in PC3 cells when treated with ABT-263 and the casapse-9 inhibitor (Suppl. Fig. 1). The possible participation of caspase-2 in this process was also evaluated. Caspase-2 has been suggested to participate in reactive oxygen species-mediated apoptosis [36], but the precise roles of this “orphan” caspase in cancer cell apoptosis have not been elucidated fully [37]. ABT-263 alone activated caspase-2 in PC3 cells (Fig. 5B), and this activation was slightly increased by the addition of the caspase-9 inhibitor (Fig. 6A). Inhibition of caspase-2 showed a tendency towards decreased apoptosis (only Annexin V^+^/PI^−^ (early) apoptosis) in ABT-263-treated PC3 cells co-treated with the caspase-9 inhibitor (Fig. 6 B and C). In these experiments, caspase-8, which is a main caspase in the ‘extrinsic’ apoptotic pathway, was found to participate in this apoptotic process, and the addition of a caspase-8 inhibitor clearly decreased apoptosis of ABT-263-treated PC3 cells under caspase-9 inhibition. This apoptotic inhibition by the caspase-8 inhibitor was more dramatic than that seen with the caspase-2 inhibitor. Interestingly, activation of caspase-2 has been reported to activate caspase-8, and sequential activation of caspase-2 and -8 is essential for saikosaponin A-induced apoptosis in human cancer cells [38]. Nevertheless, these lines of evidence reveal that treatment of caspase-9-inhibited prostate cancer cells with ABT-263 can trigger apoptosis mainly through activation of caspase-8.

What remains unclear is the mechanism by which caspase-8 becomes activated. ABT-263 alone activated caspase-8 in PC3 cells (Fig. 5B), and the combination of ABT-263 and caspase-9 inhibition also promoted caspase-8 activation (Fig. 6A). Because c-FLIP is known to inhibit caspase-8 activation [6], the possible involvement of c-FLIP was examined initially. The expression of c-FLIP_L_ and c-FLIP_S_ in PC3 cells was measured, but no apparent change was observed in cells treated with both the caspase-9 inhibitor and ABT-263 (Fig. 6A). It has also been reported that apoptosis can be induced in human prostate cancer cells via the interaction of Fas and FasL [34, 35]. Therefore, we tested whether PC3 cells express Fas and FasL, and whether treatment with anti-FasL blocking antibody could attenuate apoptosis of PC3 cells treated with both ABT-263 and the caspase-9 inhibitor. PC3 cells showed no Fas expression, and only a portion of the PC3 cells expressed FasL. Consistent with these findings, incubation with the anti-FasL antibody had no impact on apoptosis of PC3 cells treated with both ABT-263 and the caspase-9 inhibitor (Suppl. Fig. 2). Presently, we have no clear explanation for how caspase-8 is activated in ABT-263-treated PC3 cells under conditions of caspase-9 inhibition; further study is needed to elucidate the precise mechanism.

We tested three human prostate cancer cell lines in the current study. Compared with the other two lines, PC3 cells were less sensitive to DTX, but both ABT-263 and ABT-737 were able to sensitize PC3 cells to low-dose DTX. Of note, co-treatment with ABT-263 and the caspase-9 inhibitor increased caspase-8-dependent apoptosis not only in PC3 cells but also in LNCaP cells (Fig. 7). In contrast, co-treatment with the caspase-9 inhibitor significantly decreased apoptosis in ABT-263-treated DU145 cells. To explore possible explanations for this finding, the expression of a panel of Bcl-2 family proteins was examined in the three cell lines. DU145 cells showed no Bax expression and low Bak expression (Fig. 1D); since these are both pro-apoptotic molecules, this may account for the low sensitivity of the cells to ABT-263. A similar finding has been reported in ABT-737 and DU145 cells [24].

The therapeutic effect of combined DTX and Bcl-2 family inhibition was also examined using a xenograft mouse model. In this *in vivo* experiment, as the Bcl-2 family inhibitor, we used ABT-737, which has the same specificity of inhibition as ABT-263 yet can be administered systemically [20]. The combination therapy was found to significantly suppress PC3 tumor growth, compared with either therapy alone. These results suggest that the combination of Bcl-2 family inhibitors with DTX is effective not only *in vitro* but also *in vivo*. However, there remains a question. ABT-263 and ABT-737 are supposed to be similar agents with similar effects, however, the in vivo results in combination with DTX were different. Although we have no clear answer regarding this result at present, we suppose that this discrepancy in therapeutic efficacy could result from the difference in the administration routes of these reagents. We administered ABT-263 orally at 20 mg/kg for 5 consecutive days (on days 0, 1, 2, 3, and 4 after grouping) in combination with injections of DTX (10 mg/kg) twice (on days 1 and 3). More frequent administration of ABT-263 with higher doses could elicit a significant combination effect. We have no idea regarding the quantity of orally administered ABT-263 that would be absorbed in the intestine and have no information about its pharmacokinetics. Thus, we decided to use ABT-737, which showed similar activity *in vitro* but can be administered systemically.

Among the three cell lines tested, androgen-independent DU145 cells were more resistant to ABT-263 than the other two lines, and there was no difference in the sensitivity of androgen-dependent LNCaP and androgen-independent PC3 cells towards ABT-263. Additionally, PC3 cells were more resistant to DTX than LNCaP or DU145 cells. However, the results with LNCaP cells provided additional information on the role of p53 when these cells were treated with DTX and/or ABT-263. LNCaP cells express wild-type p53, PC3 cells do not express p53, and DU145 cells harbor a mutated form of p53 [39]. Together, these lines of evidence indicate that the sensitivity to ABT-263 and DTX seemed to be unconnected to androgen dependency and p53.

Emergence of DTX resistance in prostate cancer cells has been an important therapeutic hurdle. To this end, we established a DTX-resistant PC3 variant cell line, designated DR-PC3, in which we examined the cytotoxic effect of DTX combined with ABT-263 (Fig. 3). Although DR-PC3 cells were highly resistant to DTX, ABT-263 augmented the antitumor effect of DTX.

In summary, we investigated the sensitizing effect of the Bcl-2 family inhibitors ABT-263 and ABT-737 on the partially DTX-resistant PC3 human prostate cancer cell line. Our data indicate that these Bcl-2 inhibitors effectively enhance DTX-induced antitumor effects both *in vitro* and *in vivo*. These findings suggest that ABT-263 and ABT-737 may be promising agents for restoring DTX sensitivity to DTX-resistant human prostate cancers.

## METHODS

### Cell culture and reagents

Three human prostate cancer cell lines (LNCaP, PC3, and DU145) were obtained from the American Type Culture Collection (ATCC, Rockville, MD, USA), and maintained in RPMI 1640 medium (Sigma-Aldrich, St. Louis, MO, USA) supplemented with 10% fetal bovine serum (Sigma-Aldrich) and 20 μg/mL gentamicin (Nacalai Tesque, Kyoto, Japan) in a humidified atmosphere containing 5% CO_2_ at 37°C. PrEC is a normal prostate epithelial cell line purchased from Lonza (Walkersville, MD, USA). ABT-263 and ABT-737 were purchased from Active Biochemicals Co., Ltd (Wan Chai, Hong Kong). ABT-199 was purchased from ChemieTek (Indianapolis, IN, USA). DTX was obtained from Sigma-Aldrich and diluted in ethanol and finally in PBS with 5% ethanol. The pan-caspase inhibitor z-VAD-FMK was purchased from Enzo Life Sciences (Farmingdale, NY, USA), and the caspase-8 inhibitor z-IETD-FMK, caspase-9 inhibitor z-LEHD-FMK, and caspase-2 inhibitor z-VDVAD-FMK were purchased from R&D Systems (Minneapolis, MN, USA).

### Cell viability assays

Cell viability was evaluated using the 2-(2-methoxy-4-nitrophenyl)-3-(4-nitrophenyl)

-5-(2, 4-disulfophenyl)-2H-tetrazolium monosodium salt (WST-8) assay (Nacalai Tesque). Briefly, cells were seeded in flat-bottomed 96-well plates. At the assay endpoint (2 days post-seeding), 10 μl WST-8 solution was added to each well, and the plates were incubated for an additional 3 h. The plates were read at a wavelength of 450 nm using a microplate reader (Beckman Coulter, Brea, CA, USA).

### Establishment of a DTX-resistant PC3 variant cell line

To establish a DTX-resistant PC3 cell line, parental PC3 cells were cultured initially with 11 nM DTX and then with 50 nM DTX for over 4 months. The established DTX-resistant PC3 cell line was referred to as DR-PC3.

### *In vivo* xenograft model

Male BALB *nu/nu* mice, purchased from CLEA Japan (Tokyo, Japan), were maintained under specific pathogen-free conditions. The protocol was approved by the Committee on the Ethics of Animal Experiments of the Shimane University Faculty of Medicine (Permit Number: IZ26-212). All efforts were made to minimize suffering. Mice were inoculated in the right flank with 3×10^6^ PC3 cells and Matrigel (Japan BD Biosciences, Tokyo, Japan) at a 1:1 volume ratio in a total volume of 100 μl. On day 7, the mice were pooled and divided into four groups. On the indicated days, these PC3-bearing mice were treated with DTX and/or ABT-263 or ABT-737. As a vehicle control for DTX, 100 μl 5% ethanol PBS was injected. As a vehicle control for ABT-263 and ABT-737, 100 μl DMSO were administered. The tumor size and product of the two perpendicular diameters were measured every 3 or 4 days. Each group contained six mice.

### Transfection of siRNA

Transfection of siRNA was performed using Lipofectamine^TM^ RNAiMAX (Invitrogen, Grand Island, NY, USA) according to the manufacturer's instructions. siRNAs targeting Bcl-2 and Bcl-xL were purchased from Santa Cruz Biotechnology (Santa Cruz, CA, USA) and Invitrogen, respectively. Control siRNA (#6568) was purchased from Cell Signaling Technology (Danvers, MA, USA). The transfected cells were used for the experiments 3 days after siRNA transfection.

### Immunoblot

Cells were lysed using a mammalian protein extraction reagent (M-PER; PIERCE Scientific, Rockford, IL, USA) containing protease inhibitor cocktail (Nacalai Tesque). Equal amounts of protein were resolved on 4–12% gradient or 12% SDS-PAGE gels and then transferred to polyvinylidene fluoride membranes. After blocking the membranes, the blots were incubated with the following primary antibodies: anti-caspase-3 (9668: Cell Signaling Technology), anti-caspase-8 (M032-3; Medical and Biological Laboratories, Nagoya, Japan), anti-caspase-9 (9508; Cell Signaling Technology), anti-caspase-2 (2224; Cell Signaling Technology), anti-β-actin (BioLegend, San Diego, CA, USA) and anti-α-tubulin (Santa Cruz Biotechnology). Goat anti-rabbit or goat anti-mouse alkaline phosphatase-conjugated secondary antibodies (Invitrogen) were used to detect the primary antibodies. After washing, the membranes were incubated for 30 min at room temperature with an alkaline phosphatase-conjugated secondary antibody. Protein bands were visualized using CDP-star chemiluminescence and imaged using a LAS-4000 (FujiFilm, Tokyo, Japan).

### Flow cytometric analysis

Cell death was assessed using the Annexin V-FITC Apoptosis Detection kit (BioVision, Mountain View, CA, USA) and PI. Each caspase inhibitor (20 μM), or the same volume of DMSO as a vehicle control, was added at the initiation of culture. Analysis was performed using a FACSCalibur flow cytometer (Becton Dickinson).

### Statistical analyses

Data were evaluated statistically using an unpaired two-tailed Student's *t-*test or an ANOVA together with Bartlett's test. A *P-*value < 0.05 was considered to indicate significance.

## SUPPLEMENTARY FIGURES


