# Supplementary material for: Bcl-2 family inhibition sensitizes human prostate cancer cells to docetaxel and promotes unexpected apoptosis under caspase-9 inhibition
Source: Oncotarget. 2014 Oct 15;5(22):11399–412. doi: 10.18632/oncotarget.2550 (PMC4294332; doi:10.18632/oncotarget.2550)
Supplement: Supplementary file 1 [file oncotarget-05-11399-s001.pdf]

SUPPLEMENTARY FIGURES

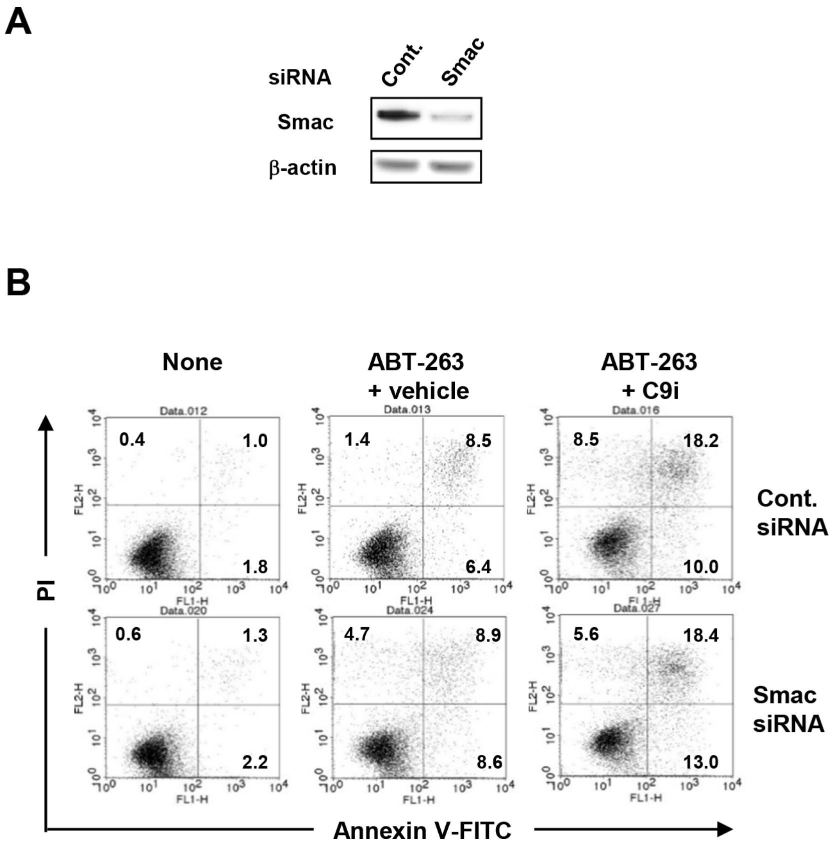

Supplementary Figure S1: The effect of ABT-263 on PC3 cells transfected with Smac siRNA. (A) PC3 cells transfected with control or Smac siRNA were analyzed for Smac expression by immunoblot. β-actin was used as the control. **(B)** siRNA-transfected PC3 cells were cultured with ABT-263 (5 μM) caspase-9 inhibitor. After 24 h, cells were stained with FITC-conjugated Annexin V and PI, and flow cytometry was performed. Representative results are shown. The numbers represent the percentages of each subset. C9i, caspase-9 inhibitor. As the vehicle control, the same volume of DMSO was added.

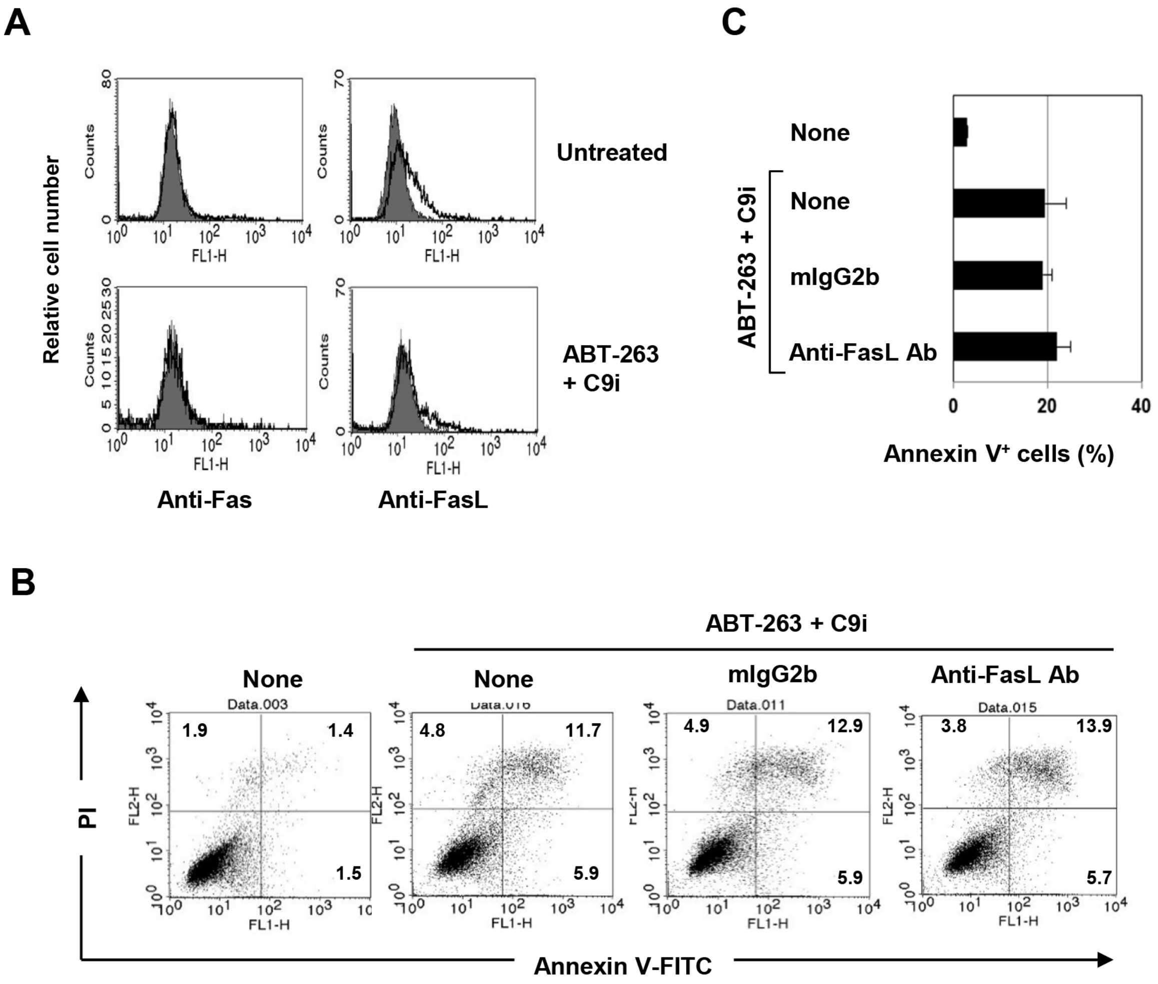

Supplementary Figure S2: Fas/FasL interaction does not contribute to the antitumor effect of ABT-263-induced caspase-8-dependent apoptosis in PC3 cells. **(A)** PC3 cells were treated with or without ABT-263 (5 µM) and the caspase-9 inhibitor (20 µM). After 24 h, flow cytometric analysis was performed using an anti-Fas or anti-FasL antibody, followed by incubation with FITC-conjugated anti-mouse IgG antibody. Gray background indicates an isotype-matched control mIgG. **(B)** PC3 cells were treated with or without ABT-263 (5 µM) and the caspase-9 inhibitor (20 µM) in the presence of anti-FasL mAb or $mIgG_{2b}$. After 24 h, cells were stained with FITC-conjugated Annexin V and PI, and flow cytometric analysis was performed. Representative results are shown. The numbers represent the percentages of each subset. **(C)** The results are shown as the means + SD of three samples. C9i, caspase-9 inhibitor.
